# Safety and Efficacy Findings From a Phase Ib/II Study of ASP‐1929 Photoimmunotherapy With Pembrolizumab in Recurrent and/or Metastatic Head and Neck Squamous Cell Carcinoma

**DOI:** 10.1002/hed.70014

**Published:** 2025-08-25

**Authors:** David M. Cognetti, Joseph M. Curry, Jennifer Johnson, Michael Kwon, Shirley Y. Su, Francisco Civantos, Coral Olazagasti, Joseph Valentino, Susanne M. Arnold, R. Bryan Bell, Michael K. Gibson, Kyle Mannion, Kathryn M. Van Abel, Katharine A. Price, Haiying Dong, Amy H. Thorne, Toshiaki Suzuki, Ann M. Gillenwater

**Affiliations:** ^1^ Department of Otolaryngology ‐ Head and Neck Surgery Thomas Jefferson University Philadelphia Pennsylvania USA; ^2^ Sidney Kimmel Comprehensive Cancer Center Thomas Jefferson University Philadelphia Pennsylvania USA; ^3^ Department of Medical Oncology Thomas Jefferson University Philadelphia Pennsylvania USA; ^4^ Department of Diagnostic Radiology, Division of Diagnostic Imaging University of Texas MD Anderson Cancer Center Houston Texas USA; ^5^ Department of Head and Neck Surgery University of Texas MD Anderson Cancer Center Houston Texas USA; ^6^ Department of Otolaryngology ‐ Head and Neck Surgery Miller School of Medicine, University of Miami Miami Florida USA; ^7^ Medical Oncology Sylvester Comprehensive Cancer Center, University of Miami Miami Florida USA; ^8^ Department of Otolaryngology ‐ Head and Neck Surgery University of Kentucky Lexington Kentucky USA; ^9^ Division of Medical Oncology University of Kentucky Lexington Kentucky USA; ^10^ Division of Surgical Oncology, Radiation Oncology, and Clinical Programs Providence Cancer Institute Portland Oregon USA; ^11^ Department of Medicine Vanderbilt University Medical Center Nashville Tennessee USA; ^12^ Department of Otolaryngology ‐ Head and Neck Surgery Vanderbilt University Medical Center Nashville Tennessee USA; ^13^ Department of Otolaryngology ‐ Head and Neck Surgery Mayo Clinic Rochester Minnesota USA; ^14^ Department of Medical Oncology Mayo Clinic Rochester Minnesota USA; ^15^ Global Data and Analytics, Rakuten Medical, Inc San Diego California USA; ^16^ Pre‐Clinical Research and Development, Rakuten Medical, Inc San Diego California USA; ^17^ Rakuten Medical, Inc San Diego California USA

**Keywords:** advanced disease, ASP‐1929 photoimmunotherapy, biomarker analysis, HNSCC, pembrolizumab

## Abstract

**Background:**

ASP‐1929 photoimmunotherapy—cetuximab conjugated to IRDye 700DX and red light (690 nm) for localized drug activation—results in rapid, selective cell killing.

**Methods:**

This phase Ib/II open‐label study evaluated ASP‐1929 photoimmunotherapy plus pembrolizumab in patients with recurrent/metastatic HNSCC (≥ 1 accessible lesion, PD‐L1 combined positive score ≥ 1, ineligible for standard locoregional therapy). Primary objectives were safety/tolerability and objective response rate (ORR). Secondary objectives included overall survival (OS) and progression‐free survival (PFS).

**Results:**

Eighteen patients (median age 63 years, 74% male) comprised the photoimmunotherapy‐evaluable population. The confirmed ORR was 27.8% (95% CI 9.7–53.5); four of five responders had complete responses (95% CI, 6.4–47.6). Median OS was 25.6 months (95% CI, 14.6–not evaluable); median PFS was 2.9 months (95% CI, 1.4–14.6). The most common serious adverse reactions were dysphagia and tongue edema (each *n* = 2; 10.5%).

**Conclusions:**

ASP‐1929 photoimmunotherapy plus pembrolizumab was generally tolerable, with promising efficacy in patients with recurrent/metastatic HNSCC.

## 1 Introduction

Head and neck squamous cell carcinoma (HNSCC) is the seventh most common cancer globally. In 2022, there were an estimated 946 000 new cases worldwide, and 482 000 deaths attributable to these cancers [1, 2]. Around 60%–70% of individuals diagnosed with primary HNSCC have locally or regionally advanced disease (stage III or IV disease) at presentation, typically characterized by large tumors with invasion of local structures, metastasis to regional lymph nodes, or both [3]. Despite aggressive therapy with surgery, radiation, and/or chemotherapy, 15%–40% of patients with advanced disease will experience recurrence [3]. As the primary source of morbidity and mortality in patients with HNSCC is locoregional progression [4], effective locoregional control remains a key therapeutic challenge.

Treatment of patients with recurrent locally advanced and/or metastatic disease can include surgery, radiation therapy, chemotherapy, targeted therapies, and immunotherapy [4, 5, 6, 7]. Recent approval of anti‐programmed cell‐death protein 1 (PD‐1) immunotherapeutic agents such as pembrolizumab and nivolumab has further advanced the treatment of patients with recurrent or metastatic HNSCC [8, 9]. Unfortunately, response rates are only approximately 20% with these agents [10].

Epidermal growth factor receptor (EGFR) is overexpressed in > 90% of patients with HNSCC, and activation of EGFR has been linked to several immunosuppressive features within the tumor microenvironment, including limited infiltration by cytotoxic T cells, suppression of antigen presentation pathways, and an increase in regulatory T‐cell (Treg) recruitment [11, 12, 13]. EGFR inhibition may reverse these immune escape mechanisms by restoring MHC class I expression, reducing Treg activity, and enhancing dendritic cell function [11].

ASP‐1929 photoimmunotherapy is a drug–device combination, in which the EGFR‐targeting agent cetuximab is conjugated to a light‐activatable dye, IRDye 700DX. Cells expressing EGFR are bound with the antibody conjugate and subsequently illuminated with red light (690 nm) for localized activation of the dye, resulting in rapid and selective cancer cell destruction [14, 15, 16]. In early‐stage clinical trials using RM‐1929, which is analogous to ASP‐1929, photoimmunotherapy demonstrated clinically meaningful activity and a manageable safety profile in patients with HNSCC [14].

Preclinical studies have shown that ASP‐1929 photoimmunotherapy reshapes the tumor microenvironment to favor antitumor immunity, increasing infiltration of effector CD8+ T cells, reducing suppressive cell populations, and synergizing with anti‐PD‐1 therapy to achieve durable tumor control [15, 16]. Photoimmunotherapy may therefore serve as a local immune‐priming modality that enhances responsiveness to checkpoint blockade.

In this paper we report results from a phase Ib/II clinical trial of ASP‐1929 photoimmunotherapy in combination with anti‐PD‐1 (pembrolizumab) treatment in patients with recurrent HNSCC that was not considered amenable to locoregional therapy (study ASP‐1929‐181; clinicaltrials.gov identifier NCT04305795).

## 2 Materials and Methods

### 2.1 Study Design and Participants

ASP‐1929‐181 is an open‐label trial of ASP‐1929 photoimmunotherapy with pembrolizumab in patients with EGFR‐expressing advanced solid tumors. The trial included a subprotocol for the treatment of patients with recurrent locally advanced or metastatic HNSCC, which is the subject of this manuscript.

Eligible patients were aged ≥ 18 years at study entry, with histologically or cytologically confirmed recurrent and/or metastatic HNSCC (primary tumor locations: oropharynx, oral cavity, hypopharynx, or larynx) that was not amendable to curative locoregional therapy, including surgical resection. Furthermore, a multi‐disciplinary group (including a surgeon and radiation oncologist) was required to agree that the patient was not a candidate for locoregional therapy. Additional inclusion criteria included tumors with known programmed cell‐death ligand 1 (PD‐L1) expression status with a combined positive score (CPS) of ≥ 1, measurable disease per modified Response Evaluation Criteria in Solid Tumors (RECIST) version 1.1, and Eastern Cooperative Oncology Group performance status of 0 or 1. Eligibility for photoimmunotherapy required at least one EGFR‐expressing tumor lesion that was accessible to light illumination. Tumor accessibility was determined by the treating investigator based on lesion visibility, location, and depth relative to surrounding anatomical structures on physical examination and contrasted computed tomography (CT) imaging. An appropriate illumination technique (e.g., frontal or cylindrical diffuser) was selected accordingly. Although there were no strict thresholds for tumor thickness, deeper lesions required careful consideration of accessibility for complete tumor illumination. No symptoms or local complications were required for inclusion.

Patients were excluded if they had a primary tumor site in the nasopharynx or had had prior systemic therapy for recurrent and/or metastatic disease within the preceding 6 months. Prior treatment with anti‐PD‐1 or anti‐PD‐L1 agents was not permitted. Radiation therapy, or any other non‐systemic therapy, within 4 weeks prior to study entry was prohibited. Chronic systemic steroid therapy (> 10 mg daily prednisone or equivalent) or any other immunosuppressive therapy in the 2 weeks before study entry was not permitted. Patients with active autoimmune disease, known or active central nervous system metastases, and/or carcinomatous meningitis were excluded.

### 2.2 Procedures

**FIGURE 1:**
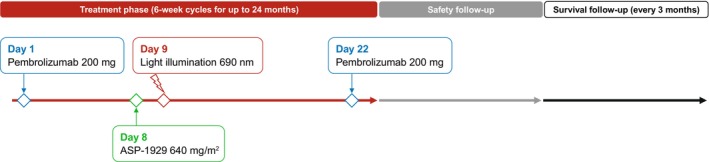
Study design. [Color figure can be viewed at wileyonlinelibrary.com]

The study design is summarized in Figure 1. Pembrolizumab 200 mg was given as a 30‐min infusion on days 1 and 22 of every 6‐week cycle. Patients could receive a single intravenous infusion of ASP‐1929 640 mg/m^2^ administered over 2 h on day 8 of each 6‐week cycle, followed by light illumination 24 (± 4) h later, on day 9. Fiber‐optic devices (frontal diffusers, cylindrical diffusers, or a combination of both) were used to direct red light and illuminate the tumor. Prior to each ASP‐1929 infusion, patients were premedicated with diphenhydramine or other antihistamines per the investigational site's standard practice.

Treatment with ASP‐1929 continued for up to 24 months unless a complete response (CR), disease progression not appropriate for further photoimmunotherapy, or unacceptable toxicity occurred. ASP‐1929 photoimmunotherapy was delayed for any grade ≥ 3 adverse events attributed to ASP‐1929 photoimmunotherapy, with the following exceptions: pain that could be managed with supportive care and analgesia; dysphagia if nutrition could be maintained through supportive care, nasogastric/gastrostomy, or jejunostomy tube; fatigue that could be managed with supportive care; constipation that could be managed with supportive care; and dehydration that could be managed with supportive care.

ASP‐1929 photoimmunotherapy treatment could also be delayed for other clinical conditions that precluded treatment in the opinion of the investigator; for example, delayed wound healing.

If the ASP‐1929‐photoimmunotherapy was delayed for > 8 weeks (more than one cycle), further ASP‐1929 photoimmunotherapy was permanently discontinued in that patient. Resumption of treatment could occur only after discussion with the study medical monitor (or designee). Irrespective of dose delays, tumor assessments were performed and other secondary endpoint data were collected as described in the subprotocol. Patients who discontinued ASP‐1929 photoimmunotherapy because of toxicity could continue with pembrolizumab therapy as a single agent and continue study assessments.

No modification of ASP‐1929 photoimmunotherapy or light illumination was permitted.

If a patient discontinued ASP‐1929 photoimmunotherapy for lack of accessible lesions, progressive disease (PD) not amenable to further ASP‐1929 photoimmunotherapy, treatment‐related toxicity, or after achieving a CR, the patient could continue with pembrolizumab treatment alone (no additional ASP‐1929 photoimmunotherapy) and participation in the study by completing study assessment visits. If a patient experienced progression of disease but had accessible tumors that could be treated by ASP‐1929 photoimmunotherapy, additional ASP‐1929 photoimmunotherapy could be administered at the discretion of the investigator and the study medical monitor.

Patients were followed for survival status every 3 months after discontinuation of study treatment until death, loss to follow‐up, or withdrawal of consent. Patients who discontinued all study treatments but whose tumor had not yet progressed could continue study visit assessments (e.g., imaging every 6 weeks). The end‐of‐treatment visit was conducted only if the patient was not continuing with study assessments in the treatment period. Collection of survival data could continue in the event of a patient's discontinuation from study treatment.

### 2.3 Objectives and Endpoints

The primary objectives of the study were to characterize the safety and tolerability of ASP‐1929 photoimmunotherapy in combination with pembrolizumab and to assess the effect of treatment on tumor response. The secondary objective was to assess the effect of ASP‐1929 photoimmunotherapy plus pembrolizumab on survival. Exploratory objectives were to assess the pharmacokinetics and presence of anti‐drug antibodies after treatment with ASP‐1929 photoimmunotherapy and to characterize the mechanism of anticancer activity of treatment.

Primary endpoints were treatment‐emergent adverse events (TEAEs), serious TEAEs, and objective response rate (ORR). TEAEs were coded according to the Medical Dictionary for Regulatory Activities, version 25.0, with severity grading according to National Cancer Institute Common Terminology Criteria for Adverse Events, version 5.0. Causality of all adverse events (AEs) was assessed independently by the investigators as related to ASP‐1929, pembrolizumab, or photoimmunotherapy. TEAEs and serious TEAEs were collected until 30 days after the last dose of study treatment. TEAEs that occurred on the same day as the ASP‐1929 infusion and were considered to be related to ASP‐1929 were assessed by the investigator to determine if they were infusion reactions. Immune‐related AEs were collected until 90 days after the last dose of pembrolizumab.

The ORR was defined as the proportion of patients with confirmed overall tumor response of CR or partial response (PR) per modified RECIST 1.1 as assessed by investigators. Modifications to RECIST v1.1 specified that “target” lesions must be both measurable by RECIST and treated with ASP‐1929 photoimmunotherapy. “Non‐target” lesions were defined as RECIST‐measurable lesions that were inaccessible to photoimmunotherapy illumination and were not treated, or lesions that were not RECIST‐measurable but were accessible to illumination and were treated with ASP‐1929 photoimmunotherapy (see Supporting Information Methods).

Secondary endpoints were: overall survival (OS), defined as the time from first dose of study treatment to death due to any cause; progression‐free survival (PFS), defined as the time from first dose of study treatment until death or PD per modified RECIST 1.1; and duration of response (DOR), defined as the time from first CR or PR to PD per modified RECIST 1.1. Exploratory endpoints included: pharmacokinetics of ASP‐1929; presence of neutralizing antibodies and anti‐drug antibodies; IR700 free dye and total antibody pharmacokinetics; changes in intratumoral and systemic immunologic biomarkers across innate and adaptive cell types and processes induced by the combination of pembrolizumab with ASP‐1929 photoimmunotherapy; and changes in the immunologic contexture in the tumor microenvironment (cell type, cell density, and cell spatial orientation) induced by the combination of pembrolizumab with ASP‐1929 photoimmunotherapy.

### 2.4 Pharmacokinetics Analysis

Eligible patients were those who received ASP‐1929 and had at least one evaluable post‐dose pharmacokinetics measurement. Samples were collected prior to ASP‐1929 infusion and at 15 min, 4 h, 24 h, 192 h, and 336 h after the end of infusion.

Pharmacokinetic parameters were derived by non‐compartmental analysis methods for all patients included in the pharmacokinetics analysis set using PKNCA R Package version 1.0 using actual sampling times. The terminal phase rate constant (λ_z_) was calculated by linear regression of at least three data points in the terminal phase of the concentration–time profile with R^2^_adj_ ≥ 0.8 in which the first time point had to be greater than t_max_. No values of λ_z_ and other λ_z_‐related parameters (e.g., t_½_, AUC_inf_) were reported for concentration profiles that did not meet these criteria. If the extrapolated portion of AUC_inf_ from the last time point to infinity for a patient was > 20%, no values of λ_z_‐related parameters were reported, and corresponding footnotes were included.

To enable comparison of pharmacokinetic profiles of ASP‐1929 monotherapy with combination ASP‐1929 and pembrolizumab therapy, a population pharmacokinetics analysis was performed using global non‐linear mixed‐effects modeling software (NONMEM, version 7.5; ICON, Dublin, Ireland) to generate concentrations of ASP‐1929 monotherapy at matching nominal times.

### 2.5 Biomarker Analysis

Tumor biopsies were analyzed for changes in effector T cells using multiplex immunofluorescence for immune markers. Changes in PD‐L1 expression (CPS and tumor proportion score) were measured with the Dako PD‐L1 IHC 22c3 pharmDx kit (Agilent Dake IHC, Santa Clara, CA). Changes in EGFR expression were assessed using the H‐score with Roche Ventana CONFIRM anti‐EGFR (3C6) Primary Antibody staining (Roche Diagnostics, Indianapolis, IN).

Bulk RNA sequencing of formalin‐fixed paraffin‐embedded tissue sections evaluated gene expression changes across treatment cycles. Tumor mutational burden, EGFR mutation status, and microsatellite instability were assessed using whole exome sequencing. Peripheral blood was analyzed for immune cell subsets at baseline in responders versus non‐responders using flow cytometry.

### 2.6 Statistical Analysis

The planned sample size for the HNSCC substudy was 26 patients. Sample size determination was based on a target ORR of 36%, which would achieve an 83% precision with a half confidence interval of 20%.

Efficacy endpoints were analyzed for the photoimmunotherapy‐evaluable population, defined as patients who received at least one dose of ASP‐1929 and illumination plus pembrolizumab. The ORR was estimated with 95% confidence intervals (CIs) based on the Clopper–Pearson method. The median OS was estimated using Kaplan–Meier product limit estimates with CIs estimated using log–log transformation of variance and additionally displayed using Kaplan–Meier plots. Similar analysis methods were applied to PFS and DOR.

Safety endpoints were descriptively analyzed for the treated population, defined as patients who received any study treatment (ASP‐1929, photoimmunotherapy, or pembrolizumab).

Analyses were performed using SAS version 9.4 (SAS institute, Cary, NC).

Biomarker analyses were performed using GraphPad Prism 10.2.3 (GraphPad Software, Boston, MA). Genes ranked by logFC values were used in a gene set enrichment analysis to identify significantly activated or suppressed genomic pathways (adjusted *p*‐value < 0.001) using the gseGO (GO biological process terms only) function in the clusterProfiler R package (version 4.8.3).

## 3 Results

Between November 2020 and April 2022, 32 patients at seven centers in the United States were screened, 22 of whom were eligible for inclusion (Figure S1). The most common reasons for ineligibility among the 10 screen failures were diagnosis and/or treatment for additional malignancy within 2 years prior to study day 1 (*n* = 2), uncontrolled intercurrent illness that would limit compliance with study requirements (*n* = 2), and CPS < 1 as determined by a Clinical Laboratory Improvement Amendments‐certified and/or US Food and Drug Administration‐approved test (*n* = 2). Three patients withdrew from the study, resulting in 19 enrolled patients. Patients were predominantly male (*n* = 14; 74%) and White (*n* = 14; 74%) and had a primary tumor in the oropharynx (*n* = 9; 47%) with no distant metastasis (*n* = 18; 95%); demographic and clinical characteristics are summarized in Table 1. Three of nine patients with a primary tumor in the oropharynx were human papillomavirus‐positive.

**TABLE 1:** Patient demographics and clinical characteristics at baseline.

Characteristic	Enrolled population (*N* = 19)
Median age (range), years	63 (44–90)
< 65 years, *n* (%)	11 (57.9)
≥ 65 years, *n* (%)	8 (42.1)
Sex, *n* (%)	
Female	5 (26.3)
Male	14 (73.7)
Race, *n* (%)	
White	14 (73.7)
Asian	1 (5.3)
Not reported	4 (21.1)
ECOG performance status, *n* (%)	
0	8 (42.1)
1	11 (57.9)
Primary tumor location, *n* (%)	
Oropharynx	9 (47.4)
Oral cavity	4 (21.1)
Larynx	3 (15.8)
Nasal cavity	2 (10.5)
Other^a^	1 (5.3)
Target lesion size, cm	
*N* ^b^	18
Mean (SD)	3.38 (1.31)
Median (range)	3.4 (1.2–5.5)
Distant metastasis, *n* (%)	
Yes	1 (5.3)
No	18 (94.7)
Nodal involvement, *n* (%)	
Yes	6 (31.6)
No	13 (68.4)
TNM stage, *n* (%)	
T1	1 (5.3)
T2	2 (10.5)
TX	16 (84.2)
N0	4 (21.1)
N2	2 (10.5)
N2B	1 (5.3)
NX	12 (63.2)
M0	18 (94.7)
M1	1 (5.3)
HPV status, *n* (%)	
Positive	5 (26.3)
Negative	9 (47.4)
Unknown/not done	3 (15.8)/2 (10.5)
CPS scoring, *n* (%)	
≥ 1% and < 20%	10 (52.6)
≥ 20%	9 (47.4)
Prior surgery, *n* (%)	17 (89.5)
Prior radiotherapy, *n* (%)	18 (94.7)
Prior systemic therapy, *n* (%)^c^	10 (52.6)

Abbreviations: CPS, combined positive score; ECOG, Eastern Cooperative Oncology Group; HPV, human papilloma virus; SD, standard deviation; TNM, tumor, node, metastasis.

^a^ Right posterior oral tongue.

^b^ One patient did not receive photoimmunotherapy and consequently had no photoimmunotherapy‐treated lesion measurements.

^c^ As part of curative treatment (adjuvant, neoadjuvant, chemoradiation) administered for primary HNSCC.

Eighteen patients received both pembrolizumab and ASP‐1929. One patient did not complete photoimmunotherapy because of a cerebrovascular accident (stroke) that occurred after administration of pembrolizumab and ASP‐1929 but before the illumination procedure. Over a median survival follow‐up of 31.7 months (range, 23–40), patients received a median of 2.5 cycles of ASP‐1929 photoimmunotherapy plus pembrolizumab (number of cycles: 1, *n* = 4; 2, *n* = 5; 3, *n* = 4; 4, *n* = 2; ≥ 6, *n* = 3; range 1–8).

Diphenhydramine and/or corticosteroid premedication was administered to 18 patients. Two patients with target lesions in the oropharynx and tongue underwent a prophylactic tracheostomy concurrently with their first illumination to prevent potential complications arising from airway obstruction. Two patients had a post‐procedure tracheostomy as described below.

### 3.1 Efficacy

**FIGURE 2:**
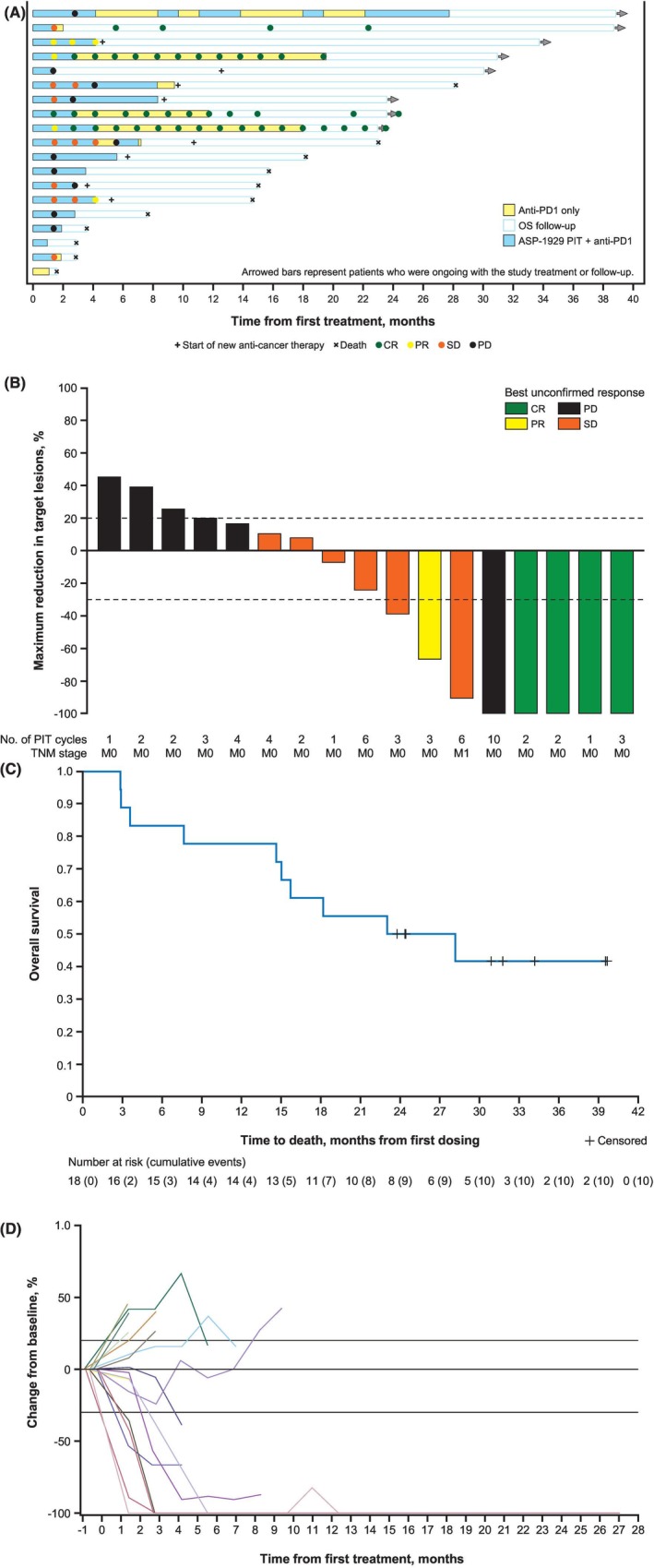
(A) Timepoint tumor response (until PD) in the treated population. (B) Best change in tumor size in the efficacy‐evaluable population. Patient 1 ultimately achieved a CR but was deemed to have PD according to RECIST version 1.1 because of new lesions early in treatment that were subsequently treated. (C) Overall survival in photoimmunotherapy‐evaluable patients treated with ASP‐1929 photoimmunotherapy. (D) Spaghetti plot of percentage change from baseline in sum of diameters of target lesions over time (investigator‐assessed). The treated population included patients who received one or both study treatments (part A); the photoimmunotherapy‐evaluable population included patients who received both study treatments for at least one cycle (part C); the efficacy‐evaluable population included patients who had at least one post‐baseline tumor assessment (parts B and D). Data cutoff: April 30, 2024. CR, complete response; OS, overall survival; PD, progressive disease; PD‐1, programmed cell death protein 1; PIT, photoimmunotherapy; PR, partial response; RECIST, Response Evaluation Criteria in Solid Tumors; SD, stable disease; TNM, tumor, node, metastasis staging system.

Eighteen patients received a minimum of one complete treatment with both pembrolizumab and ASP‐1929 photoimmunotherapy and were considered evaluable for response. This interim evaluation was performed with a data cutoff date of April 30, 2024, at which time all 19 patients had discontinued the study treatment. The median number of treatment cycles (ASP‐1929 plus pembrolizumab) was 2.5 (range, 1–8); the median number of pembrolizumab cycles was 3 (range, 1–17); and the median duration of exposure to ASP‐1929 was 18.0 weeks (range, 6–123). Efficacy data are shown in Table 2 and Figure 2.

**TABLE 2:** Summary of tumor response in photoimmunotherapy‐evaluable patients (*N* = 18)^a^.

Measure	*n* (%)	95% CI
Objective response rate, *n* (%)	5 (27.8)	9.7–53.5
Disease control rate, *n* (%)	11 (61.1)	35.7–82.7
Best overall response, *n* (%)		
Complete response	4 (22.2)	6.4–47.6
Partial response	1 (5.6)	0.1–27.3
Stable disease	6 (33.3)	13.3–59.0
Progressive disease	6 (33.3)	13.3–59.0
Not evaluable	1 (5.6)	0.1–27.3
Median overall survival, months	25.6	14.6–NE
Kaplan–Meier estimates for rates, %		
6 months	83.3	56.8–94.3
12 months	77.8	51.1–91.0
18 months	61.1	35.3–79.2
24 months	50.0	25.9–70.1
Median progression‐free survival, months	2.9	1.4–14.6
Kaplan–Meier estimates for rates, %		
6 months	34.3	13.5–56.5
12 months	34.3	13.5–56.5
18 months	27.5	9.0–49.9

*Note*: Cutoff date: April 30, 2024.

Abbreviations: CI, confidence interval; NE, not evaluable.

^a^ All patients who received at least one ASP‐1929 infusion and illumination.

**FIGURE 3:**
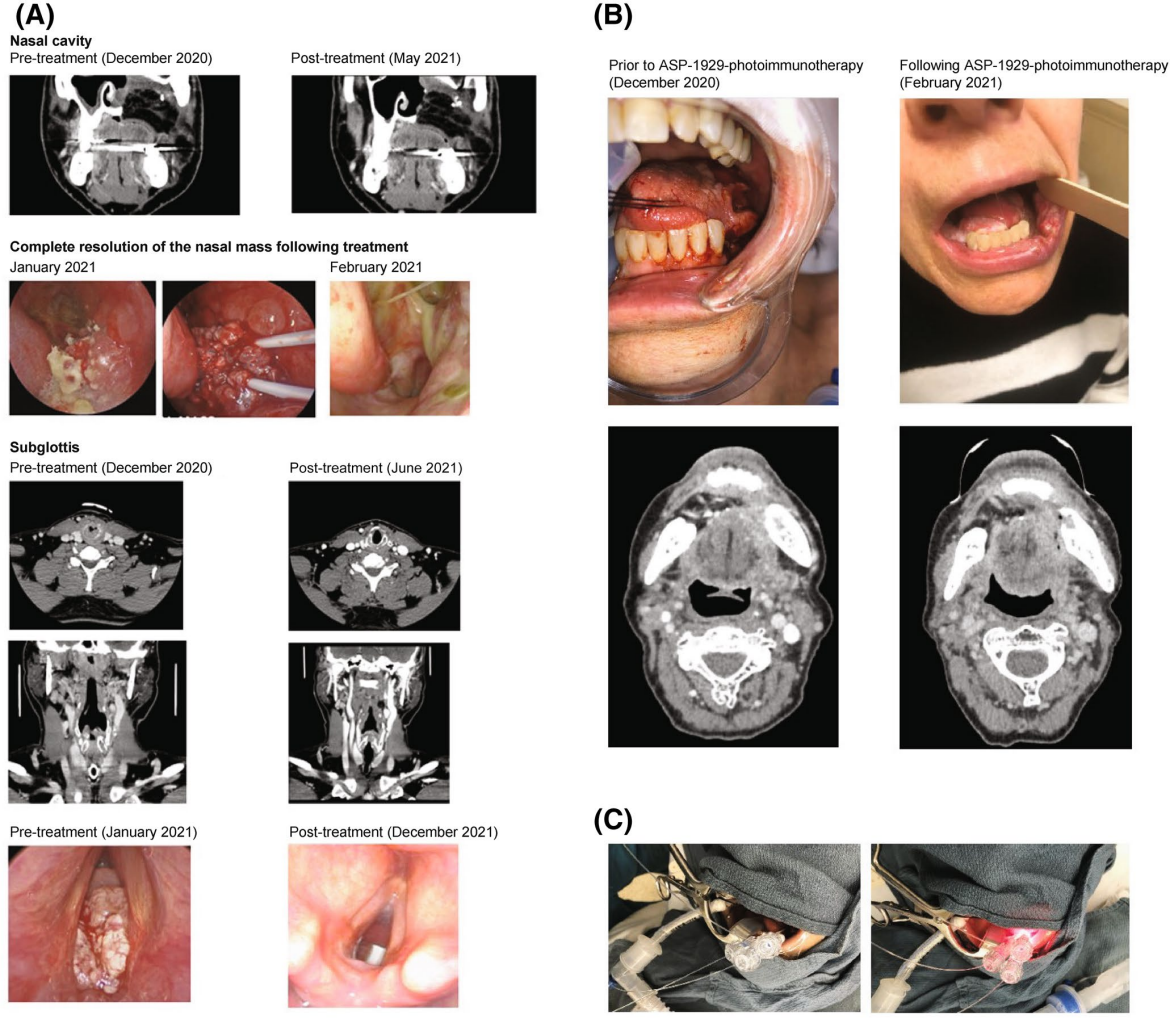
Examples of patients with durable responses after ASP‐1929 photoimmunotherapy and pembrolizumab. (A) Patient case #1: The figure shows the clinical course of a 44‐year‐old man with a history of sinonasal squamous cell carcinoma arising from a maxillary sinus inverting papilloma. He was initially diagnosed with inverting papilloma with high‐grade dysplasia treated in the community with endoscopic resection. His disease recurred 3 months later, and biopsy confirmed inverting papilloma with high‐grade dysplasia. He was treated again in the community with endoscopic surgery with positive margins and radiotherapy. Twelve months after completing radiotherapy, he presented with a recurrent mass in the maxillary sinus, confirmed to be squamous cell cancer, and a lesion in the subglottis read as papilloma with dysplasia. He underwent a total maxillectomy with free flap reconstruction followed by re‐irradiation. Three months after completion of radiotherapy, he then underwent a laser resection of the subglottic lesion with final pathology showing non‐keratinizing squamous cell cancer arising from a squamous papilloma. Simultaneously, he developed recurrences in the nasal cavity. Following one cycle of combination study treatment, areas treated with ASP‐1929 photoimmunotherapy showed no evidence of disease (NED). However, new disease sites emerged in the nasopharynx and trachea, leading to an overall response evaluation of progressive disease (PD) in accordance with modified RECIST v1.1. The patient received an additional two cycles of ASP‐1929 photoimmunotherapy and pembrolizumab combination therapy and achieved a complete response (CR). Approximately 7 months later, as a result of disease progression while on pembrolizumab monotherapy, ASP‐1929 photoimmunotherapy was resumed for an additional two cycles, again reverting the patient to CR. Over the course of 2 years, the patient received a total of 10 cycles of ASP‐1929 photoimmunotherapy. At the last office visit prior to data cut, the patient had NED for approximately 15 months. Represented as Patient #1 in Figure 1. (B) Patient case #2: This 57‐year‐old woman presented with recurrence of invasive keratinizing HNSCC in 2020. She had a prior history of HNSCC of the right floor of mouth (2018), which developed in the setting of left‐sided lichen planus, that had been monitored since 2014. Prior interventions included multiple surgeries and radiation therapy with relatively rapid cadence for recurrence. The patient received one cycle of the study combination regimen (ASP‐1929 photoimmunotherapy and pembrolizumab) beginning in January 2021, and a second cycle was initiated with pembrolizumab. The patient developed biopsy‐negative ulceration on the lower lip, initially considered to be due to photoimmunotherapy. Upon further examination of the photoimmunotherapy‐treated area, and in consideration of the increasing severity and distribution of ulceration, an autoimmune cheilitis diagnosis was rendered. The patient was withdrawn from study treatment and given steroid therapy. In March 2021, the patient was considered to have a CR, with NED observed at follow‐up. At the time of data cut (April 30, 2024), the patient remained NED. Represented as Patient #2 in Figure 1. (C) ASP‐1929 photoimmunotherapy procedure (diffuser placement and illumination) in Patient #2.

Four confirmed CRs and one confirmed PR were observed, for a confirmed ORR of 27.8% (95% CI, 9.7–53.5). One patient initially assessed as having stable disease (SD) on CT imaging was subsequently determined to have achieved a CR at a follow‐up scan (patient case #2). A further six patients had SD, resulting in a disease control rate of 61.1% (95% CI, 35.7–82.7). Time to confirmed response and DOR were assessed in the five confirmed responders. Responses were rapid in many cases: the median time to response was 1.4 months (95% CI, 1.4 to not evaluable). Responses were durable, with DOR of 16.9+, 18.0+, 22.1+, and 23.0+ months in patients with CR and 2.8+ months in the patient with a PR; neither PD nor death were observed in responding patients at data cutoff. The median DOR was not reached. One patient had an early best response of PD (modified RECIST v1.1) based on the appearance of new non‐target lesions, but continued combination treatment for approximately 28 months per the investigator's judgment, and ultimately achieved a CR (Figure 3).

The median OS in the photoimmunotherapy‐evaluable population at data cutoff was 25.6 months (95% CI, 14.6 to not evaluable; Figure 2C). The median PFS was 2.9 months (95% CI, 1.4–14.6).

Examples of treatment responses are shown in Figure 3: the first, patient case #1, was a 44‐year‐old man with a history of sinonasal squamous cell carcinoma previously treated with surgery and radiation, followed by rapid recurrence. He received ASP‐1929 photoimmunotherapy and pembrolizumab after recurrence at multiple sites including the nasal cavity, nasopharynx, and subglottis. The second, patient case #2, was a 57‐year‐old woman with recurrence of invasive keratinizing HNSCC in the oral cavity. Both patients ultimately achieved a CR.

### 3.2 Safety

All patients experienced at least one TEAE (Table S1). The most frequent all‐grade TEAEs were fatigue, oral pain, and constipation (Table 3). TEAEs of grade ≥ 3 severity were reported in 14 patients (74%; Table S1). There were no fatal events. Two patients experienced grade 4 events: tumor hemorrhage related to advanced disease and laryngeal edema related to photoimmunotherapy.

**TABLE 3:** TEAEs occurring in > 15% of patients in the treated population (*N* = 19).

Event	Any grade, *n* (%)	Grade ≥ 3, *n* (%)
Any event	19 (100.0)	14 (73.7)
Fatigue	11 (57.9)	0
Oral pain	10 (52.6)	1 (5.3)
Constipation	7 (36.8)	0
Neck pain	6 (31.6)	0
Headache	6 (31.6)	0
Hypothyroidism	6 (31.6)	0
Infusion‐related reaction	5 (26.3)	0
Muscular weakness	5 (26.3)	2 (10.5)
Dysphagia	4 (21.1)	2 (10.5)
Nausea	4 (21.1)	0
Stomatitis	4 (21.1)	0
Tongue edema	4 (21.1)	1 (5.3)
Rash maculopapular	4 (21.1)	0
Ear pain	4 (21.1)	0
Localized edema	4 (21.1)	0
Application site pain	3 (15.8)	0
Diarrhea	3 (15.8)	0
Vomiting	3 (15.8)	0
Edema peripheral	3 (15.8)	0
Aspiration	3 (15.8)	1 (5.3)
Cough	3 (15.8)	0
Productive cough	3 (15.8)	1 (5.3)
Dehydration	3 (15.8)	0
Weight decreased	3 (15.8)	2 (10.5)
Insomnia	3 (15.8)	0

Abbreviation: TEAEs, treatment‐emergent adverse events.

Serious TEAEs are summarized in Table S2. Five patients experienced serious TEAEs related to ASP‐1929 photoimmunotherapy: one had grade 3 dysphagia; one had grade 3 dysphagia and tongue edema that required post‐procedure tracheostomy; one had grade 3 tongue edema and grade 2 hypoxia; one had grade 4 laryngeal edema that required post‐procedure tracheostomy; and one had grade 3 facial pain.

Infusion reactions, all related to ASP‐1929, were reported for five patients and consisted of non‐serious grade 1 or 2 events (rash, itching, back pain, dyspnea, hyperhidrosis, stomach cramps/ache, tightness of throat, fever, myalgia diarrhea, headache, chills, nausea, and vomiting). There were no infusion reactions greater than grade 2 in severity (Table S2).

Immune‐related AEs associated with anti‐PD‐1 therapy occurred in three patients who experienced one or more of the following events: cheilitis, thyroiditis, hypothyroidism, blisters, paronychia, temperature intolerance, paresthesia, and fatigue. All were non‐serious grade 1 or 2 AEs, except for one patient with grade 3 serious cheilitis (Table S2).

Three patients developed non‐serious AEs related to both ASP‐1929 and pembrolizumab. Two had fatigue and headache during the first treatment cycle, one of whom also developed blisters during the second cycle and paresthesia during the third cycle. Another patient experienced hyperhidrosis during the second cycle. All were grade 1 events.

Three patients experienced AEs that led to treatment discontinuation: grade 2 acute kidney injury related to pembrolizumab; grade 3 cheilitis attributed to pembrolizumab; and grade 3 stroke deemed not likely related to any study treatment. No discontinuations were related to ASP‐1929 photoimmunotherapy.

### 3.3 Pharmacokinetics

Ten patients (male, *n* = 9; median age 56 years) were included in the interim pharmacokinetic analysis, all of whom had received at least one dose of ASP‐1929. Serum pharmacokinetic parameters for patients treated with single‐dose ASP‐1929 (640 mg/m^2^ intravenous infusion) are shown in Table 4. Single‐dose evaluations were performed as ASP‐1929 washed out completely between the 6‐week dosing intervals.

**TABLE 4:** Summary of serum pharmacokinetic parameters of single‐dose ASP‐1929 640 mg/m^2^ intravenous infusion.

PK parameter	No. of patients	Arithmetic mean (arithmetic CV%)
AUC_inf_, μg/mL*h	9	13 650 (24.59)
AUC_last_, μg/mL*h	9	13 070 (24.35)
CL, L/h	9	0.09708 (28.77)
C_max_, μg/mL	10	328.5 (13.77)
t_max_, h^a^	10	2.4 (2.2, 7.1)
t_½_, h	9	46.81 (24.27)
V_z_, L	9	6.313 (23.95)

Abbreviations: AUC_inf_, area under the concentration–time profile from time zero to infinite time; AUC_last_, area under the concentration–time profile from time zero extrapolated to the time of the last quantifiable concentration; CL, total body clearance; C_max_, maximum concentration; CV, coefficient of variation; PK, pharmacokinetics; t_½_, terminal elimination half‐life; t_max_, time until C_max_ is reached; V_z_, terminal phase volume of distribution.

^a^ Median (range).

A preliminary comparison of ASP‐1929 concentrations from the study against population pharmacokinetic‐derived ASP‐1929 concentrations from ASP‐1929 monotherapy studies at matched nominal times (data on file) suggests that combination with pembrolizumab does not affect ASP‐1929 exposure.

### 3.4 Biomarker Analysis

**FIGURE 4:**
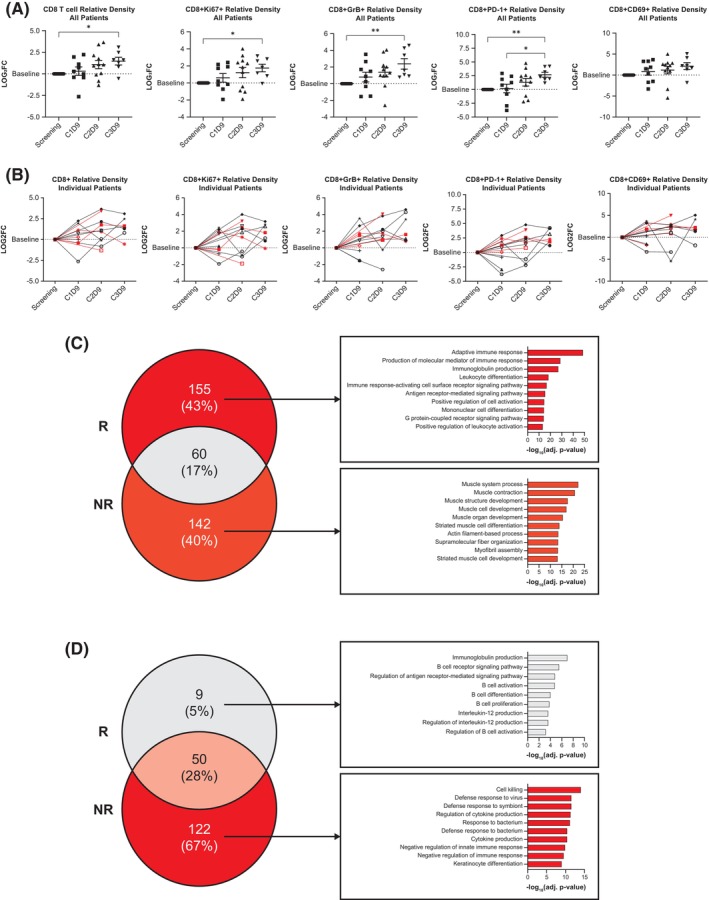
(A) Changes in CD8 T‐cell populations over time by multiplex immunofluorescence within the tumor microenvironment. (B) Changes in individual patients (black represents non‐responders, red represents responders). (C) RNAseq analysis of the top upregulated pathways in tumor biopsies at C1D9 versus screening. The Venn diagram shows common and uncommon upregulated pathways in responders (R) versus non‐responders (NR). Among the non‐common pathways, the top 10 upregulated pathways for responders or non‐responders are displayed to the right. Note that among 142 upregulated pathways in non‐responders, none are associated with immune activation. Upregulated pathways for responders suggest that priming of the immune system has occurred in response to pre‐treatment with pembrolizumab. (D) RNAseq analysis of top upregulated pathways in tumor biopsies at C2D9 versus screening, with the top nine upregulated pathways for responders and top 10 upregulated pathways for non‐responders. Note that of 122 upregulated pathways in non‐responders, only 17 are associated with immune response and are primarily negative regulators. Upregulated pathways in responders are associated with increased antitumor immunity through T‐cell priming, antibody‐dependent cell‐mediated cytotoxicity, and antibody‐dependent cellular phagocytosis [17]. [Color figure can be viewed at wileyonlinelibrary.com]

Evaluation of tumor infiltrating lymphocytes using multiplex immunofluorescence staining of patient tumor biopsies showed a strong increase in CD8+ T‐cell densities throughout treatment, suggesting an immune response with ASP‐1929 photoimmunotherapy plus pembrolizumab. All patients had increases in CD8+ T cells that were proliferating (Ki67+), cytotoxic (granzyme B+), and/or exhausted (PD‐1+); these increases were statistically significant at day 9 of cycle 3 (C3D9) compared with screening; however, there was no correlation with response (Figure 4A,B).

Bulk RNA sequencing revealed statistically significant gene expression differences between responders and non‐responders, particularly in immune‐activation pathways in responders at C1D9 (Figure 4C) and B‐cell‐related pathways at C2D9 (Figure 4D).

**FIGURE 5:**
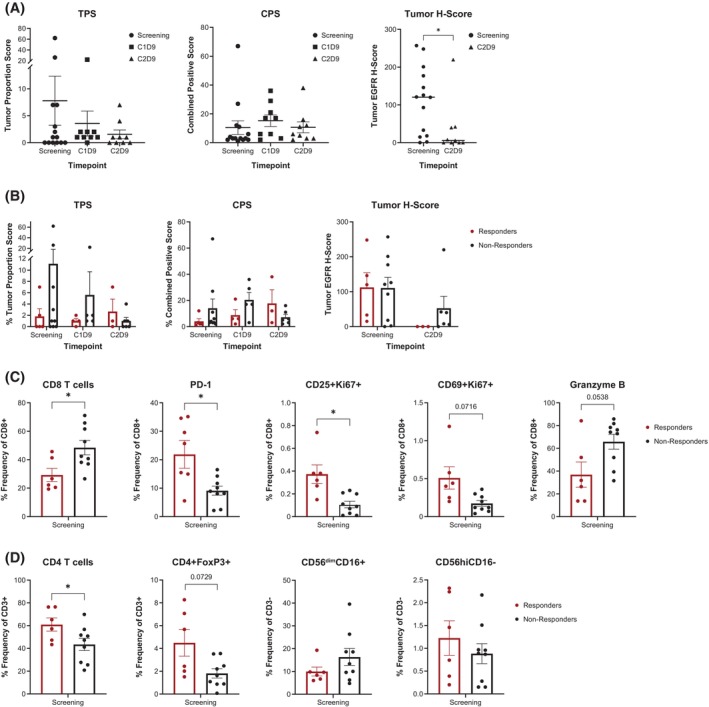
(A) TPS, CPS, and EGFR H‐score at screening and during treatment. (B) TPS, CPS, and tumor H‐score in responders and non‐responders. Differences between responders and non‐responders at baseline (screening) in (C) peripheral blood CD8 T‐cell populations, (D) CD4 T‐cell populations, and natural killer‐cell populations measured by flow cytometry. CPS, combined positive score; EGFR, epidermal growth factor receptor; PD‐1, programmed cell death protein 1; TPS, tumor proportion score. *p < 0.05. [Color figure can be viewed at wileyonlinelibrary.com]

No predictive trends were seen for PD‐L1 tumor proportion score or CPS (Figure 5A,B), EGFR expression (Figure 5A, right panel) or mutation status, tumor mutational burden, or microsatellite instability (data not shown); however, there was a significant reduction in EGFR expression across patients following ASP‐1929 photoimmunotherapy (Figure 5A, right panel). Baseline peripheral immunity analysis using flow cytometry revealed that responders exhibited statistically significant reductions in CD8+ T‐cell counts and increases in PD‐1 + CD8+ T‐cell counts compared with non‐responders (Figure 5C). Additionally, responders demonstrated statistically significant elevations in Ki67+, CD25+, and CD8+ T cells, as well as circulating CD4+ T‐cell and natural killer‐cell levels at baseline compared with non‐responders (Figure 5C,D).

## 4 Discussion

In this study, the novel approach of ASP‐1929 photoimmunotherapy in combination with pembrolizumab showed promising activity in patients with recurrent locoregional HNSCC that was not amenable to standard locoregional therapies, with a median OS of 25.6 months, ORR of 27.8%, and a disease control rate of 61.1% after a median follow‐up of 31.7 months. Responses were seen in five of 18 photoimmunotherapy‐treated patients, with SD in a further six patients.

Although cross‐study comparisons are made with caution because of differences in patient populations and study designs, the median OS of 25.6 months is promising, in view of data from other studies. Patients with CPS ≥ 1 in KEYNOTE‐048 [18] who were treated with pembrolizumab alone and pembrolizumab plus chemotherapy had OS times of 12.3 months and 13.6 months, respectively, and those in CheckMate 651 who received nivolumab plus ipilimumab had a median OS of 15.7 months [19]. Notably, KEYNOTE‐048 and CheckMate 651 had a higher proportion of patients with distant metastases compared with the present study. Additionally, both studies excluded patients with rare conditions, such as sinonasal squamous cell carcinoma, unlike the present study. As a result of the anatomical restrictions of the study population, we were unable to treat tumors close to or invading major arteries, unlike the KEYNOTE‐048 and CheckMate 651 studies. Given these limitations, the present findings should be considered exploratory and hypothesis‐generating.

Responses were rapid, with a median time to response of 1.4 months, and several responses were apparent at the first assessment. Early determination of patient response to new therapies is crucial, as it enables clinicians to quickly transition non‐responders to alternative treatments; it can take several months to determine a patient's response to standard chemotherapy or new investigational drugs. Responses were also durable, with all DORs in patients who had a CR exceeding 16 months and ongoing at data cutoff, suggesting the potential for durable clinical activity with this combination therapy. Evaluating treatment response shortly after photoimmunotherapy can be difficult, particularly when localized effects such as inflammation, necrosis, and tissue swelling occur after light activation. These changes can interfere with standard imaging interpretation and may resemble disease progression, especially when assessments are conducted early, particularly at around 6 weeks after treatment. Investigators in the present study used a combination of imaging and clinical assessment based on modified RECIST v1.1 criteria to determine candidacy for additional cycles of treatment. Where applicable, direct examination was also incorporated, particularly for tumors in accessible mucosal sites. Investigators were familiar with the expected post‐treatment tissue changes and considered these factors when evaluating response. CT imaging at a follow‐up visit was also considered when assigning a response category in one patient (patient #2), who appeared to have SD on initial imaging but was later confirmed to have had a CR. Imaging alone may not always provide a clear picture of early treatment efficacy, and clinical context and physical examination were often critical in guiding decisions about retreatment with photoimmunotherapy.

ASP‐1929 photoimmunotherapy plus pembrolizumab was generally tolerable. Although serious treatment‐related AEs were observed in almost one‐quarter of patients, these were generally manageable and their incidence was comparable with results from the completed RM‐1929 photoimmunotherapy study in 30 patients with recurrent HNSCC [14], as well as other studies using pembrolizumab in patients with relapsed or metastatic HNSCC [10, 20]. The serious TEAEs dysphagia and tongue edema are local effects of ASP‐1929 photoimmunotherapy and can also be observed among patients with recurrent HNSCC as part of the underlying disease or as a result of prior radiation therapy. The present data suggest that the combination of immuno‐oncologics with ASP‐1929 photoimmunotherapy does not increase the risks of AEs compared with either treatment administered alone. However, the potential for AEs resulting from the combination therapy warrants consideration, treatment planning, and an individualized risk–benefit assessment approach.

Analysis of pharmacokinetic parameters suggests that the addition of pembrolizumab to ASP‐1929 does not affect exposure to ASP‐1929, consistent with the observed absence of AEs of greater than grade 1 related to both ASP‐1929 and anti‐PD‐1 in this study.

Biomarker analysis revealed increased T‐cell infiltration and activation, and EGFR target engagement. Upregulated pathways in responders' biopsies at cycle 2 highlighted B‐cell proliferation and activation, suggesting ASP‐1929 photoimmunotherapy may modify B‐cell activity and the tumor microenvironment toward antitumor immunity. C1D9 gene expression changes revealed immune priming due to pembrolizumab in responders. Peripheral immunity at baseline aligned with known predictors of checkpoint inhibition response [21]. These biomarker results support the role of immune activation and B‐cell immunity in response to ASP‐1929 photoimmunotherapy plus pembrolizumab, which may be suggestive of a maintained adaptive immune response [22].

It must be acknowledged that photoimmunotherapy is a locally directed treatment, and that although targeted lesion(s) may benefit from this treatment, other lesions may occur or progress outside the field of illumination. Patient case #1 (Figure 2, Case 1) demonstrated the versatility of ASP‐1929 photoimmunotherapy plus pembrolizumab, where PD resulting from new lesions would have otherwise led to early treatment discontinuation. Despite multifocal disease in multiple head and neck sites, repeated combination treatments in this study ultimately led to a durable CR for the patient, with no evidence of disease for 15 months at the last visit. Additionally, the synergy of ASP‐1929 photoimmunotherapy plus pembrolizumab was demonstrated in the same patient when a CR achieved after three cycles of ASP‐1929 photoimmunotherapy plus pembrolizumab progressed after approximately 7 months of pembrolizumab monotherapy but was successfully converted back to a CR following two cycles of ASP‐1929 photoimmunotherapy plus pembrolizumab.

Limitations of this study include the small size of the patient population, lack of a control arm, and limitations of RECIST v1.1 assessments following photoimmunotherapy. This was a cohort of patients with locally recurrent and/or metastatic HNSCC that was considered not amendable to curative surgical resection; however, only one patient had distant metastatic disease. Enrollment into the study was closed prior to reaching the planned sample size. Although the ORR did not meet the predefined target, the observed median OS was encouraging, and no new unexpected safety signals were identified. These findings were considered sufficiently compelling evidence to inform the design of a subsequent confirmatory phase III trial in a larger, more representative population. Although RECIST v1.1 is a useful tool for tumor evaluation in systemic therapy, it has its limitations when assessing tumor response to photoimmunotherapy. Similar to other locoregional therapies such as surgery and radiation, RECIST v1.1 primarily evaluates changes in tumor size; however, photoimmunotherapy may cause localized effects such as necrosis, inflammation, edema, or eschar, which may be misinterpreted as disease progression or lack of response in radiologic imaging because of the nature and timing of assessments. Per RECIST v1.1, occurrence of new lesion(s) is indicative of progression and, therefore, of systemic therapy failure. This is not always the case for locally directed photoimmunotherapy, as illustrated with our first patient case example. Finally, subsequent anticancer therapies received after study discontinuation may have impacted OS.

## 5 Conclusion

The encouraging early efficacy outcomes, particularly OS, and tolerability profile described in this report support continued clinical investigation of ASP‐1929 photoimmunotherapy in combination with pembrolizumab. These findings provide a strong rationale for a phase III study to further evaluate this combination in a broader patient cohort.

## Author Contributions

Concept and design: David M. Cognetti, Joseph M. Curry, and Ann M. Gillenwater. Administrative support: David M. Cognetti, R. Bryan Bell, Michael K. Gibson, Susanne M. Arnold, Kathryn M. Van Abel, and Ann M. Gillenwater. Provision of study materials or patients: David M. Cognetti, Joseph M. Curry, Jennifer Johnson, Michael Kwon, Shirley Y. Su, Francisco Civantos, Coral Olazagasti, Joseph Valentino, Susanne M. Arnold, R. Bryan Bell, Michael K. Gibson, Kathryn M. Van Abel, Katharine A. Price, Kyle Mannion, Michael K. Gibson, and Ann M. Gillenwater. Collection and assembly of data: David M. Cognetti, Joseph Curry, Shirley Y. Su, Joseph Valentino, Kathryn M. Van Abel, Katharine A. Price, Amy H. Thorne, and Ann M. Gillenwater. Data analysis and interpretation: David M. Cognetti, Joseph M. Curry, Haiying Dong, Amy H. Thorne, Toshiaki Suzuki, and Ann M. Gillenwater. Manuscript writing: All authors. Final approval of manuscript: All authors. Accountable for all aspects of the work: All authors.

## Ethics Statement

The study protocol was approved by institutional review boards at each participating site. The study was conducted in accordance with the general ethical principles outlined in the Declaration of Helsinki and applicable laws and regulations. All patients provided written informed consent.

## Consent

The authors have nothing to report.

## Conflicts of Interest

David M. Cognetti: Travel and Advisory Role: Rakuten Medical, Inc. Joseph M. Curry: No relevant conflicts to disclose; research agreement with Regeneron. Jennifer Johnson: No conflicts to disclose. Michael Kwon: No conflicts to disclose. Shirley Y. Su: Stock and Other Ownership Interests: REsmed, CSL, Intellia Therapeutics (not relevant to this research). Francisco Civantos: No conflicts to disclose. Coral Olazagasti: Consulting or Advisory Role: Novocure, AstraZeneca, Catalyst, Pfizer, MJH life sciences. Joseph Valentino: No conflicts to disclose. Susanne M. Arnold: Consulting or Advisory Role: Penn State Cancer Institute; Research funding: AstraZeneca; Merck Sharp & Dohme; Exelixis; Ohio State University; National Cancer Institute; Kura Oncology; Syneos Health; Kinnate Biopharma; Ellipses Pharma; Gilead Sciences; Incyte; Lilly; BeiGene; Lisata Therapeutics; Bicara Therapeutics; Fortrea. R. Bryan Bell: Consulting or Advisory Role: Merck, EMD Serono, Bright Peak Therapeutics, Regeneron, Adela Bio. Michael K. Gibson: No conflicts to disclose. Kyle Mannion: No conflicts to disclose. Kathryn M. Van Abel: Patents, Royalties, Other Intellectual Property: Disclosed invention of Mayo Clinic intellectual property licensed to Exact Sciences (Madison, WI) with potential to earn royalties, paid to Mayo Clinic; Consulting or Advisory Role: Intuitive Surgical. Katharine A. Price: Consulting or Advisory Role: InSitu Biologics, PDS Biotechnology. Haiying Dong: Employment: Rakuten Medical, Inc. Amy Thorne: Employment: Rakuten Medical, Inc. Toshiaki Suzuki: Employment: Rakuten Medical, Inc. Ann M. Gillenwater: Consulting or Advisory Role: Rakuten Medical, Inc.

## Supporting information


**Data S1:** Supporting Information.

## Data Availability

The Rakuten Medical, Inc. (RMI) data sharing policy is compliant with ICMJE guidelines. RMI will consider reasonable requests for clinical data from qualified researchers. Data may be shared, at RMI's sole discretion, with external researchers whose proposed use of the data has been approved. Complete deidentified data will be eligible for sharing 2 years after completion of the relevant study. Before release of any data, the recipient must enter into a data sharing agreement with RMI, after which the deidentified data sets can be accessed within a secured portal.

## References

[1] F. Bray , M. Laversanne , H. Sung , et al., “Global Cancer Statistics 2022: GLOBOCAN Estimates of Incidence and Mortality Worldwide for 36 Cancers in 185 Countries,” CA: A Cancer Journal for Clinicians 74, no. 3 (2024): 229–263. 38572751 10.3322/caac.21834

[2] H. Sung , J. Ferlay , R. L. Siegel , et al., “Global Cancer Statistics 2020: GLOBOCAN Estimates of Incidence and Mortality Worldwide for 36 Cancers in 185 Countries,” CA: A Cancer Journal for Clinicians 71, no. 3 (2021): 209–249. 33538338 10.3322/caac.21660

[3] L. Q. M. Chow , “Head and Neck Cancer,” New England Journal of Medicine 382, no. 1 (2020): 60–72. 31893516 10.1056/NEJMra1715715

[4] Z. S. Zumsteg , M. Luu , E. J. Yoshida , et al., “Combined High‐Intensity Local Treatment and Systemic Therapy in Metastatic Head and Neck Squamous Cell Carcinoma: An Analysis of the National Cancer Data Base,” Cancer 123, no. 23 (2017): 4583–4593. 28817183 10.1002/cncr.30933 PMC5745815

[5] M. H. Cohen , H. Chen , S. Shord , et al., “Approval Summary: Cetuximab in Combination With Cisplatin or Carboplatin and 5‐Fluorouracil for the First‐Line Treatment of Patients With Recurrent Locoregional or Metastatic Squamous Cell Head and Neck Cancer,” Oncologist 18, no. 4 (2013): 460–466. 23576486 10.1634/theoncologist.2012-0458 PMC3639534

[6] D. E. Johnson , B. Burtness , C. R. Leemans , V. W. Y. Lui , J. E. Bauman , and J. R. Grandis , “Head and Neck Squamous Cell Carcinoma,” Nature Reviews Disease Primers 6, no. 1 (2020): 92. 10.1038/s41572-020-00224-3 PMC7944998 33243986

[7] J. Rubin Grandis , M. F. Melhem , W. E. Gooding , et al., “Levels of TGF‐Alpha and EGFR Protein in Head and Neck Squamous Cell Carcinoma and Patient Survival,” Journal of the National Cancer Institute 90, no. 11 (1998): 824–832. 9625170 10.1093/jnci/90.11.824

[8] J. P. Machiels , C. René Leemans , W. Golusinski , C. Grau , L. Licitra , and V. Gregoire , “Squamous Cell Carcinoma of the Oral Cavity, Larynx, Oropharynx and Hypopharynx: EHNS‐ESMO‐ESTRO Clinical Practice Guidelines for Diagnosis, Treatment and Follow‐Up,” Annals of Oncology 31, no. 11 (2020): 1462–1475. 33239190 10.1016/j.annonc.2020.07.011

[9] National Comprehensive Cancer Network , “Head and Neck Cancers. Version 2.2024. 2023”, https://www.nccn.org/professionals/physician_gls/pdf/head‐and‐neck.pdf.

[10] B. Burtness , K. J. Harrington , R. Greil , et al., “Pembrolizumab Alone or With Chemotherapy Versus Cetuximab With Chemotherapy for Recurrent or Metastatic Squamous Cell Carcinoma of the Head and Neck (KEYNOTE‐048): A Randomised, Open‐Label, Phase 3 Study,” Lancet 394, no. 10212 (2019): 1915–1928. 31679945 10.1016/S0140-6736(19)32591-7

[11] R. M. Srivastava , S. C. Lee , P. A. Andrade Filho , et al., “Cetuximab‐Activated Natural Killer and Dendritic Cells Collaborate to Trigger Tumor Antigen‐Specific T‐Cell Immunity in Head and Neck Cancer Patients,” Clinical Cancer Research 19, no. 7 (2013): 1858–1872. 23444227 10.1158/1078-0432.CCR-12-2426 PMC3640274

[12] R. Chaudhary , R. J. C. Slebos , L. C. Noel , et al., “EGFR Inhibition by Cetuximab Modulates Hypoxia and IFN Response Genes in Head and Neck Squamous Cell Carcinoma,” Cancer Research Communications 3, no. 5 (2023): 896–907. 37377902 10.1158/2767-9764.CRC-22-0443 PMC10202124

[13] K. Okuyama , T. Naruse , and S. Yanamoto , “Tumor Microenvironmental Modification by the Current Target Therapy for Head and Neck Squamous Cell Carcinoma,” Journal of Experimental & Clinical Cancer Research 42, no. 1 (2023): 114. 37143088 10.1186/s13046-023-02691-4 PMC10161653

[14] D. M. Cognetti , J. M. Johnson , J. M. Curry , et al., “Phase 1/2a, Open‐Label, Multicenter Study of RM‐1929 Photoimmunotherapy in Patients With Locoregional, Recurrent Head and Neck Squamous Cell Carcinoma,” Head & Neck 43, no. 12 (2021): 3875–3887. 34626024 10.1002/hed.26885 PMC9293150

[15] M. A. Hsu , S. M. Okamura , C. D. De Magalhaes Filho , et al., “Cancer‐Targeted Photoimmunotherapy Induces Antitumor Immunity and Can Be Augmented by Anti‐PD‐1 Therapy for Durable Anticancer Responses in an Immunologically Active Murine Tumor Model,” Cancer Immunology, Immunotherapy 72, no. 1 (2023): 151–168. 35776159 10.1007/s00262-022-03239-9 PMC9813181

[16] T. Nagaya , J. Friedman , Y. Maruoka , et al., “Host Immunity Following Near‐Infrared Photoimmunotherapy Is Enhanced With PD‐1 Checkpoint Blockade to Eradicate Established Antigenic Tumors,” Cancer Immunology Research 7, no. 3 (2019): 401–413. 30683733 10.1158/2326-6066.CIR-18-0546 PMC8237708

[17] R. Tan , M. Nie , and W. Long , “The Role of B Cells in Cancer Development,” Frontiers in Oncology 12 (2022): 958756. 36033455 10.3389/fonc.2022.958756 PMC9403891

[18] K. J. Harrington , B. Burtness , R. Greil , et al., “Pembrolizumab With or Without Chemotherapy in Recurrent or Metastatic Head and Neck Squamous Cell Carcinoma: Updated Results of the Phase III KEYNOTE‐048 Study,” Journal of Clinical Oncology 41, no. 4 (2023): 790–802. 36219809 10.1200/JCO.21.02508 PMC9902012

[19] R. I. Haddad , K. Harrington , M. Tahara , et al., “Nivolumab Plus Ipilimumab Versus EXTREME Regimen as First‐Line Treatment for Recurrent/Metastatic Squamous Cell Carcinoma of the Head and Neck: The Final Results of CheckMate 651,” Journal of Clinical Oncology 41, no. 12 (2023): 2166–2180. 36473143 10.1200/JCO.22.00332 PMC10115555

[20] A. G. Sacco , R. Chen , F. P. Worden , et al., “Pembrolizumab Plus Cetuximab in Patients With Recurrent or Metastatic Head and Neck Squamous Cell Carcinoma: An Open‐Label, Multi‐Arm, Non‐Randomised, Multicentre, Phase 2 Trial,” Lancet Oncology 22, no. 6 (2021): 883–892. 33989559 10.1016/S1470-2045(21)00136-4 PMC12140401

[21] H. J. An , H. J. Chon , and C. Kim , “Peripheral Blood‐Based Biomarkers for Immune Checkpoint Inhibitors,” International Journal of Molecular Sciences 22, no. 17 (2021): 9414. 34502325 10.3390/ijms22179414 PMC8430528

[22] B. A. Helmink , S. M. Reddy , J. Gao , et al., “B Cells and Tertiary Lymphoid Structures Promote Immunotherapy Response,” Nature 577, no. 7791 (2020): 549–555. 31942075 10.1038/s41586-019-1922-8 PMC8762581

