# Supplementary material for: Safety and Efficacy Findings From a Phase Ib/II Study of ASP‐1929 Photoimmunotherapy With Pembrolizumab in Recurrent and/or Metastatic Head and Neck Squamous Cell Carcinoma
Source: Head Neck. 2025 Aug 25;48(1):160–74. doi: 10.1002/hed.70014 (PMC12703558; doi:10.1002/hed.70014)
Supplement: Supplementary file 1 — Data S1: Supporting Information. [file HED-48-160-s001.docx]

Safety and Efficacy Findings from a Phase Ib/II Study of ASP-1929 Photoimmunotherapy with Pembrolizumab in Recurrent and/or Metastatic Head and Neck Squamous Cell Carcinoma

David M. Cognetti, MD^1,2^; Joseph M. Curry, MD^1,2^; Jennifer Johnson, MD^2,3^; Michael Kwon, MD^4^; Shirley Y. Su, MBBS^5^; Francisco Civantos, MD^6^; Coral Olazagasti, MD^7^; Joseph Valentino, MD PhD^8^; Susanne M. Arnold, MD^9^; R. Bryan Bell, MD^10^; Michael K. Gibson, MD^11^; Kyle Mannion, MD^12^; Kathryn M. Van Abel, MD^13^; Katharine A. Price, MD^14^; Haiying Dong, MS^15^; Amy H. Thorne, PhD^16^; Toshiaki Suzuki, MD^17^; Ann M. Gillenwater, MD^18^

# SUPPLEMENTARY APPENDIX

## Supplementary Methods

### Modified Response Criteria in Solid Tumours, Version 1.1 (RECIST 1.1) for Head and Neck Squamous Cell Cancer Substudy

For this substudy, a modified RECIST v1.1^1^ was used. The modifications to RECIST v1.1 include the following.

- For the purposes of this study, “target” lesions were both measurable by RECIST and treated with ASP-1929 photoimmunotherapy. RECIST-measurable lesions that were not accessible to photoimmunotherapy illumination and were not treated should be considered to be “non-target” lesions.
- All accessible lesions were treated with ASP-1929 photoimmunotherapy, regardless of RECIST-measurable status. Lesions that were not RECIST-measurable or did not meet minimum size criteria for RECIST but were accessible and treated with photoimmunotherapy illumination were also to be considered “non-target” lesions.

**SUPPLEMENTARY TABLE S1.**Summary of TEAEs

| Event | (N = 19) |
| --- | --- |
| Any TEAE | 19 (100) |
| Highest grade of TEAE |  |
| 1 | 2 (10.5) |
| 2 | 3 (15.8) |
| 3 | 12 (63.2) |
| 4 | 2 (10.5) |
| 5 | 0 |
| Serious TEAE | 12 (63.2) |
| Serious TEAE related to any study treatment^a^ | 6 (31.6) |
| Serious TEAE related to ASP-1929 photoimmunotherapy | 5 (26.3) |
| Serious TEAE related to anti-PD-1 | 1 (5.3) |
| TEAE leading to discontinuation of ASP-1929 photoimmunotherapy^b^ | 2 (10.5) |
| TEAE leading to discontinuation of anti-PD-1 | 3 (15.8) |
| TEAE leading to death | 0 |
| Immune-related AE | 3 (15.8) |

Abbreviations: AE, adverse event; TEAE, treatment-emergent adverse event.

^a^ASP-1929, illumination, or anti-PD-1.

^b^One patient with non-treatment-related cerebrovascular accident and one with cheilitis (treatment-related).

SUPPLEMENTARY TABLE S2. Key TEAEs (n=19)

| Preferred term | No. of patients (%) |
| --- | --- |
| Serious TEAEs | 12 (63.2) |
| Dysphagia | 2 (10.5) |
| Tongue edema | 2 (10.5) |
| Cheilitis | 1 (5.3) |
| Atrial fibrillation | 1 (5.3) |
| Cerebrovascular accident | 1 (5.3) |
| Cheilitis | 1 (5.3) |
| Hypertension | 1 (5.3) |
| Muscular weakness | 1 (5.3) |
| Pneumonia | 1 (5.3) |
| Pneumonia aspiration | 1 (5.3) |
| Subcutaneous abscess | 1 (5.3) |
| Tachycardia | 1 (5.3) |
| Tumor hemorrhage | 1 (5.3) |
| Urinary retention | 1 (5.3) |
| AESI |  |
| Fistula | 0 |
| Other non-serious TEAEs |  |
| Infusion reactions to ASP-1929 | 5 (26.3) |
| Immune-related AEs to anti-PD-1 | 3 (15.8) |
| Hypothyroidism | 1 (5.3) |
| Thyroiditis | 1 (5.3) |
| Cheilitis | 1 (5.3) |
| Fatigue | 1 (5.3) |
| Temperature intolerance | 1 (5.3) |
| Paronychia | 1 (5.3) |
| Paresthesia | 1 (5.3) |
| Blister | 1 (5.3) |

Abbreviations: AEs, adverse events; AESI, AE of special interest; TEAEs, treatment-emergent adverse events.

AEs are coded using the Medical Dictionary for Regulatory Activities, version 25.0.

SUPPLEMENTARY FIGURE S1. Patient flow (CONSORT diagram).


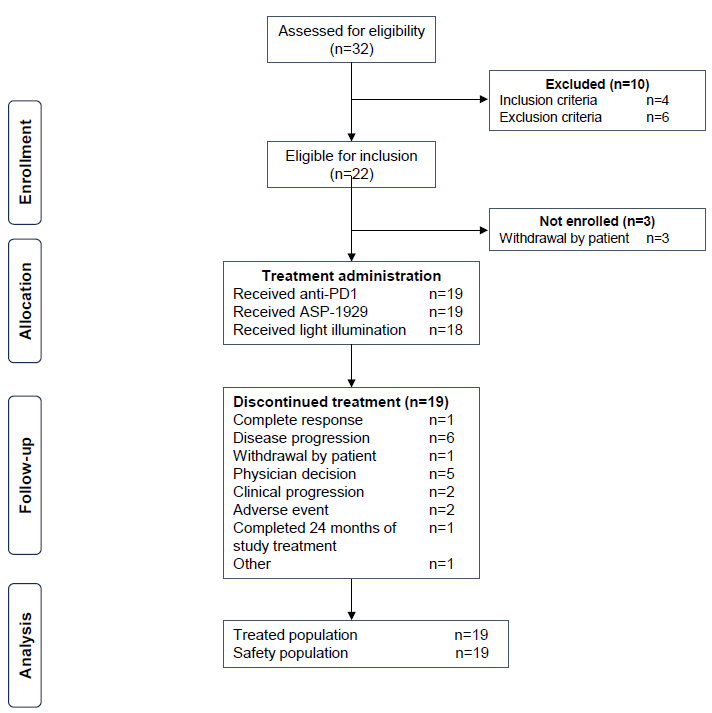


Reference

1. Eisenhauer EA, Therasse P, Bogaerts J, et al. New response evaluation criteria in solid tumours: revised RECIST guideline (version 1.1). *Eur J Cancer* 2009;45(2):228-247.
